# Beneficial effects of early empirical administration of appropriate antimicrobials on survival and defervescence in adults with community-onset bacteremia

**DOI:** 10.1186/s13054-019-2632-1

**Published:** 2019-11-20

**Authors:** Ching-Chi Lee, Chung-Hsun Lee, Chao-Yung Yang, Chih-Chia Hsieh, Hung-Jen Tang, Wen-Chien Ko

**Affiliations:** 1 Department of Internal Medicine, Madou Sin-Lau Hospital, No. 20, Lingzilin, 72152, Madou Dist., Tainan City, Taiwan; 2 Graduate Institute of Medical Sciences, College of Health Sciences, Chang Jung Christian University, Tainan, Taiwan; 3 Department of Internal Medicine, National Cheng Kung University Hospital, College of Medicine, National Cheng Kung University, No. 138, Sheng Li Road, 70403 Tainan, Taiwan; 4 Department of Medicine, National Cheng Kung University Medical College, Tainan, Taiwan; 5 Department of Emergency Medicine, National Cheng Kung University Hospital, No. 138, Sheng Li Road, 70403 Tainan, Taiwan; 6 Division of Infectious Disease, Department of Medicine, Chi-Mei Medical Center, No. 901, Chung-Hwa Road, Yung-Kang City, 710 Tainan, Taiwan; 7 Department of Health and Nutrition, Chia Nan University of Pharmacy and Science, Tainan, Taiwan

**Keywords:** Empirical, Antimicrobial therapy, Community-onset, Bloodstream infection, Febrile, Mortality

## Abstract

**Background:**

Bloodstream infections are associated with high morbidity and mortality, both of which contribute substantially to healthcare costs. The effects of early administration of appropriate antimicrobials on the prognosis and timing of defervescence of bacteremic patients remain under debate.

**Methods:**

In a 6-year retrospective, multicenter cohort, adults with community-onset bacteremia at the emergency departments (EDs) were analyzed. The period from ED arrival to appropriate antimicrobial administration and that from appropriate antimicrobial administration to defervescence was regarded as the time-to-appropriate antibiotic (TtAa) and time-to-defervescence (TtD), respectively. The primary study outcome was 30-day mortality after ED arrival. The effects of TtAa on 30-day mortality and delayed defervescence were examined after adjustment for independent predictors of mortality, which were recognized by a multivariate regression analysis.

**Results:**

Of the total 3194 patients, a TtAa-related trend in the 30-day crude (*γ* = 0.919, *P* = 0.01) and sepsis-related (*γ* = 0.909, *P* = 0.01) mortality rate was evidenced. Each hour of TtAa delay was associated with an average increase in the 30-day crude mortality rate of 0.3% (adjusted odds ratio [AOR], 1.003; *P* < 0.001) in the entire cohort and 0.4% (AOR, 1.004; *P* < 0.001) in critically ill patients, respectively, after adjustment of independent predictors of 30-day crude mortality. Of 2469 febrile patients, a TtAa-related trend in the TtD (*γ* = 0.965, *P* = 0.002) was exhibited. Each hour of TtAa delay was associated with an average 0.7% increase (AOR, 1.007; *P* < 0.001) in delayed defervescence (TtD of ≥ 7 days) after adjustment of independent determinants of delayed defervescence. Notably, the adverse impact of the inappropriateness of empirical antimicrobial therapy (TtAa > 24 h) on the TtD was noted, regardless of bacteremia severity, bacteremia sources, or causative microorganisms.

**Conclusions:**

The delay in the TtAa was associated with an increasing risk of delayed defervescence and 30-day mortality for adults with community-onset bacteremia, especially for critically ill patients. Thus, for severe bacteremia episodes, early administration of appropriate empirical antimicrobials should be recommended.

## Background

Empirical administration of appropriate antimicrobials to septic or infective individuals has remained challenging because of the increased antibiotic resistance in both hospitals and communities [1] as well as atypical presentations in the specific population, such as the elderly [2] and human immunodeficiency virus-infected adults [3]. Particularly in septic patients presenting with a critical illness, inappropriate empirical therapy is associated with increased mortality [4, 5]. Generally, bloodstream infections lead to high morbidity and mortality and substantially contribute to healthcare costs [6]. However, whether appropriate initial antimicrobial administration has beneficial effects on the outcome of patients with bloodstream infections remains debated [4, 7–11].

To shorten the hospital stay, the timing of defervescence in response to antimicrobial therapy must be addressed. Numerous studies on bacteremia [12] and pneumonia [13, 14] found a significant association between defervescence timing and administration of antimicrobial classes. However, for bloodstream infections, the beneficial effect of rapid administration of appropriate antimicrobial therapy on defervescence remains unclear. Accordingly, we hypothesized that timely appropriate antimicrobial therapy heralds a favorable outcome and speedy defervescence, and a large cohort of adults with community-onset bacteremia was studied.

## Methods

### Study design

This retrospective cohort study was conducted at the emergency departments (EDs) of two hospitals in southern Taiwan: a university-affiliated medical center with 1200 beds and another teaching hospital with 800 beds. The patient (aged ≥ 18 years) with community-onset bacteremia from January 2010 to December 2015 was included. The study was approved by the institutional review board of two study hospitals and reported by the format recommended by STROBE (Strengthening the Reporting of Observational Studies in Epidemiology).

### Data collection

During the study period, blood cultures sampled at the EDs were screened for bacterial growth by a computer database. Clinical information of adults with bacteremia was retrieved from medical charts (electronic and paper records). For the patients with multiple bacteremic episodes, only the first episode of each patient was included. Patients with community-onset bacteremia were eligible after the exclusion of those with hospital-onset bacteremia, contaminated blood cultures, bacteremia diagnosed prior to visiting the ED, incomplete clinical information of covariates listed in the predetermined case-record form, or uncertain fatality within 30 days after bacteremia onset. A predetermined record form was used to collect clinical variables, including age, vital signs at the EDs and during hospitalization, comorbidities and comorbidity severity (graded by the McCabe classification), image studies, the duration and type of antimicrobials and antipyretics administered, bacteremia sources, surgical record, the duration of hospital stay, bacteremia severity (evaluated by the Pitt bacteremia score), and patient outcomes. Furthermore, to study the time-to-defervescence (TtD), only febrile patients (with tympanic body temperature ≥ 38.3 °C) were included, and afebrile or hypothermic patients during the first 24 h after ED arrival, those with any dose of systemic steroid or antipyretics during antimicrobial therapy, those with inadequate source control, and those with uncertain date of defervescence were excluded. Two of the authors were randomly assigned to review electronic medical records and paper records. The primary study endpoint was the crude mortality within 30 days after ED arrival. The TtD, the duration of hospitalization, and the 30-day sepsis-related and crude mortality rate were regarded as the patient outcome.

### Definitions

A patient aged ≥ 65 years was regarded as the elderly [15]. Community-onset bacteremia indicates that the place of onset of bacteremic episodes is the community, which includes long-term healthcare facility- and community-acquired bacteremia, as previously described [12, 16]. Polymicrobial bacteremia was defined as the isolation of more than one microbial species from a single bacteremic episode. Blood cultures with the growth of potentially contaminating pathogens were considered to be contaminated, based on the previous criteria [17].

As previously described [12, 16], antimicrobial therapy was considered appropriate when all of the following criteria were fulfilled: (i) the route and dosage of antimicrobial agents were administered as recommended in the Sanford Guide [18]; (ii) bacteremic pathogens were in vitro susceptible to the administrated antimicrobial agent based on the contemporary breakpoints of the Clinical and Laboratory Standards Institute (CLSI) [19]. The time-to-appropriate antibiotic (TtAa) was defined as the period between the bacteremia onset (i.e., ED triage) and administration of appropriate antimicrobials [16, 20].

Body temperature was measured within the first 24 h after ED arrival; if fever or hypothermia was observed more than once in one patient, the extreme temperature was recorded. Hypothermia was defined as temporal body temperature of ≤ 36.0 °C. Defervescence was defined as an afebrile state in which tympanic body temperature maintained at < 37.0 °C for at least 24 h [12, 21], and the TtD as the period between the initiation of appropriate antimicrobial administration and the onset of defervescence. The bacteremia severity was graded, according to a Pitt bacteremia score using a previously validated scoring system based on vital signs, use of intravenous vasopressors, mental status, receipt of mechanical ventilation, and cardiac arrest during the first 24 h after ED arrival [12, 16]. A high Pitt bacteremia score (≥ 4) was indicative of a critical illness, and a Pitt bacteremia score of < 4 was indicative of a non-critical illness. Comorbidities were defined as described previously [22], and malignancies included hematological malignancies and solid tumors. The prognosis of comorbid diseases was assessed by a previous established McCabe classification [23]. The removal of infected hardware, drainage of infected fluid collections, or resolution of obstruction in biliary or urinary tracts was referred as adequate source control for complicated bacteremia, as previously defined [24–26]. Crude mortality was used to define the death from all causes, and sepsis-related mortality was defined based on the circumstance of death, as determined by the review of the available recorded observation and septic parameters.

### Microbiological methods

Blood cultures were incubated in a BACTEC 9240 instrument (Becton Dickinson Diagnostic Systems, Sparks, MD, USA) for 5 days at 35 °C. Bacterial species was identified by means of the Vitek 2 system (bioMe’rieux, Durham, NC), and antimicrobial susceptibility was determined by the disk diffusion method, based on contemporary CLSI standards [19]. To ensure the appropriateness of antimicrobial therapy for each bacteremia episode, aerobic isolates in the study period were prospectively stored. If empirical antibiotics were not included in the initial susceptibility panel, their susceptibility was measured after the inclusion in the study.

### Statistical analyses

Statistical analyses were performed using the Statistical Package for the Social Sciences for Windows (Version 23.0; Chicago, IL, USA). Clinical parameters were compared by the Fisher exact test or Pearson chi-square test for categorical variables and the independent *t* or Mann–Whitney *U* test for continuous variables. Trends in the TtAa and patient outcomes were analyzed by the equivalent linear-by-linear association test (i.e., Pearson’s correlation). To study the independent impact of TtAa delay on 30-day mortality or delayed defervescence, the variables with a *P* value of < 0.05 in the univariate analysis were included in a stepwise and backward multivariable logistic regression model. A *P* value of less than 0.05 was considered significant.

## Results

### Demographics and clinical characteristics of the entire cohort

**Fig. 1:**
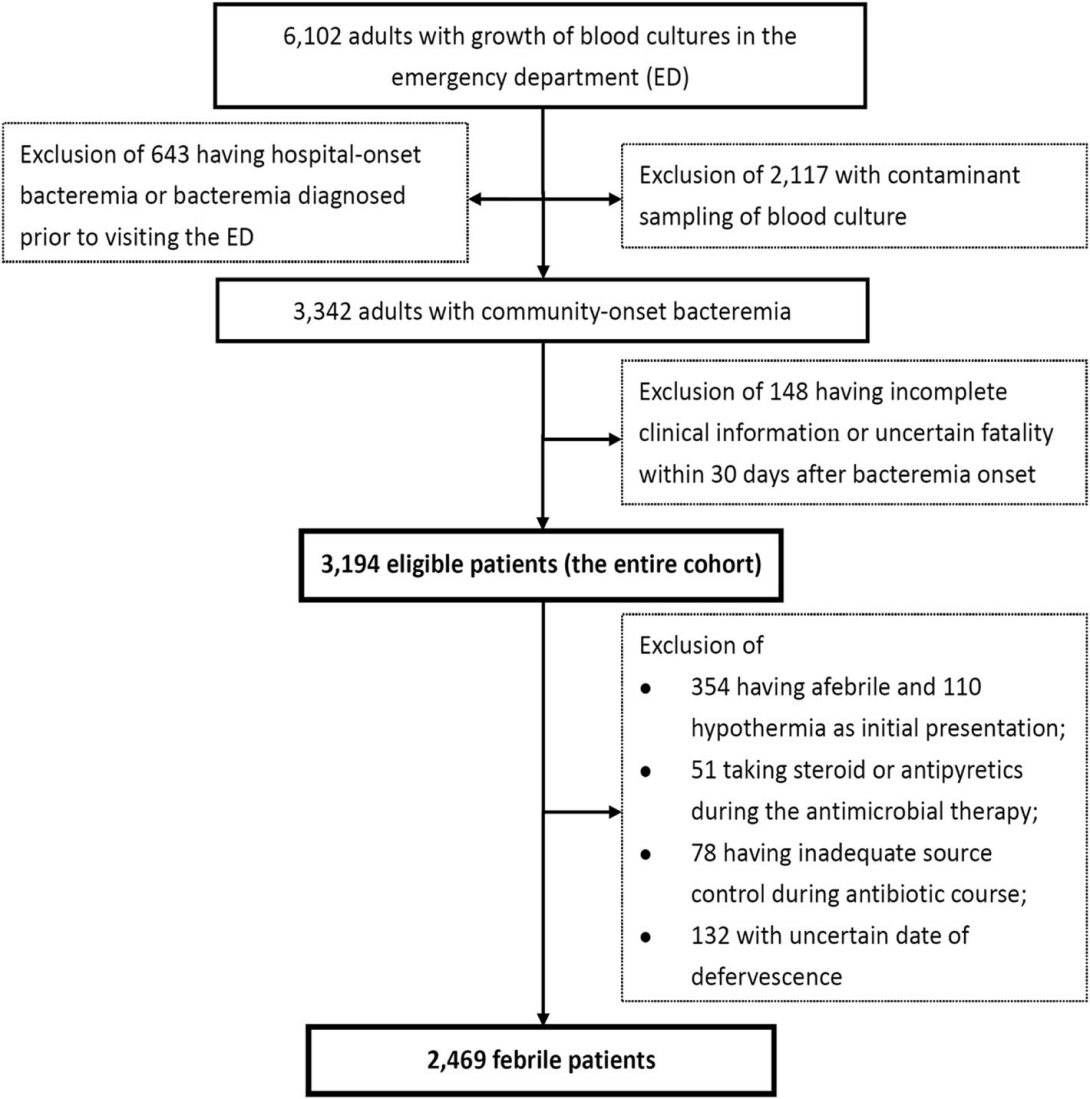
Flowchart of patient selection

Of 6102 adults with bacterial growth in blood cultures, 3194 eligible patients with community-onset bacteremia were included, according to the inclusion and exclusion criteria (Fig. 1). Of 3194 patients, their mean age was 67.6 years and 1625 (50.9%) were male (Table 1). Overall, 2922 patients (91.5%) had hospitalization through the EDs, 105 (3.3%) died during the ED stay, and 167 (5.2%) discharged from the EDs and followed up in the outpatient clinics. Fever at the ED stay was noted in 2469 (77.3%) patients. The median (interquartile range, IQR) of the ED stay, intravenous antimicrobial administration, and hospitalization was 15.6 (5.9–26.7) h, 9 (5–16) days, and 11 (6–18) days, respectively. The proportion of critically ill patients at ED arrival was 20.2% (646 patients). The 30-day crude mortality rate in critically and non-critically ill patients was 46.7% (302) and 14.8% (472), respectively.

**Table 1:** Patient demography and clinical characteristics of the study cohort, and the patients surviving at or dying within 30 days after bacteremia onset*

Variables	Patient number (%)			*P* value**
	Total *n* = 3194	Death *n* = 472	Survival *n* = 2722	
Patient demography				
Age, years, mean ± SD	*67.6 ± 16.2*	*69.9 ± 15.0*	*67.3 ± 16.4*	*0.001*
Gender, male	*1625 (50.9)*	*283 (60.0)*	*1342 (49.3)*	*< 0.001*
Nursing-home residents	*178 (5.6)*	*57 (12.1)*	*121 (4.4)*	*< 0.001*
Time-to-appropriate antibiotic, h, median (IQR)	*2 (1–8)*	*2 (1–26)*	*2 (1–7)*	*< 0.001*
Inadequate source control during antibiotic therapy	*98 (3.1)*	*25 (5.3)*	*73 (2.7)*	*0.002*
Severity-of-illness markers at ED				
Pitt bacteremia score ≥ 4	*646 (20.2)*	*302 (64.0)*	*344 (12.6)*	*< 0.001*
ICU admission through ED	*328 (10.3)*	*118 (25.0)*	*210 (7.7)*	*< 0.001*
Major bacteremia sources				
Urinary tract	*1034 (32.4)*	*51 (10.8)*	*983 (36.1)*	*< 0.001*
Pneumonia	*473 (14.8)*	*176 (37.3)*	*297 (10.9)*	*< 0.001*
Intra-abdominal	383 (12.0)	61 (12.9)	322 (11.8)	0.50
Skin and soft-tissue	307 (9.6)	45 (9.5)	262 (9.6)	0.95
Biliary tract	*272 (8.5)*	*22 (4.7)*	*250 (9.2)*	*0.001*
Primary bacteremia	263 (8.2)	42 (8.9)	221 (8.1)	0.57
Bone and joint	117 (3.7)	10 (2.1)	107 (3.9)	0.05
Vascular-line	110 (3.4)	17 (3.6)	93 (3.4)	0.84
Infective endocarditis	101 (3.2)	17 (3.6)	84 (3.1)	0.55
Liver abscess	*101 (3.2)*	*7 (1.5)*	*94 (3.5)*	*0.02*
Fatal comorbidities (McCabe classification)	*811 (25.4)*	*228 (48.3)*	*583 (21.4)*	*< 0.001*
Major comorbidities				
Hypertension	1563 (48.9)	216 (45.8)	1347 (49.5)	0.14
Diabetes mellitus	1187 (37.2)	158 (33.5)	1029 (37.8)	0.07
Malignancies	*951 (29.8)*	*228 (48.3)*	*723 (26.6)*	*< 0.001*
Neurological diseases	*735 (23.0)*	*130 (27.5)*	*605 (22.2)*	*0.01*
Chronic kidney diseases	587 (18.4)	86 (18.2)	501 (18.4)	0.92
Liver cirrhosis	*399 (12.5)*	*92 (19.5)*	*307 (11.3)*	*< 0.001*
Coronary artery diseases	309 (9.7)	48 (10.2)	261 (9.6)	0.69
Urological disorder	250 (7.8)	30 (6.4)	220 (8.1)	0.20

*ED* emergency department, *ICU* intensive care unit, *IQR* interquartile range, *SD* standard deviation

*Data are number (%) of patients unless otherwise stated

**Indicates the comparison between fatal patients and survivors. Italics indicates statistical significance, i.e., a *P* value of < 0.05

In the entire cohort (Table 1), the five leading sources of bacteremia were urinary tract infections (1034 patients, 32.4%), pneumonia (473, 14.8%), intra-abdominal infections (383, 12.0%), skin and soft-tissue infections (307, 9.6%), and biliary tract infections (272, 8.5%). The five major comorbidities included hypertension (1563 patients, 48.9%), diabetes mellitus (1187, 37.2%), malignancies (951, 29.8%), neurological diseases (735, 23.0%), and chronic kidney diseases (587, 18.4%). Patients with complicated bacteremia requiring source control accounted for 20.4% (653 patients), and those without adequate source control was merely 2.4% (78).

### Bacteremic isolates and susceptibilities

Because of 306 (9.6%) episodes of polymicrobial bacteremia, a total of 3583 causative microorganisms from 3194 patients were collected. Gram-positive aerobes majorly included *Staphylococcus aureus* (418 isolates, 11.7%), *Streptococcus* species (396, 11.1%), and *Enterococcus* species (117, 3.3%). The five leading Gram-negative aerobes were *Escherichia coli* (1332 isolates, 37.2%), *Klebsiella* species (497, 13.9%), *Pseudomonas* species (129, 3.6%), *Enterobacter* species (96, 2.6%), and *Proteus* species (88, 2.5%).

Cefazolin, cefuroxime, cefotaxime, ceftazidime, cefepime, ertapenem, ampicillin/sulbactam, piperacillin/tazobactam, or levofloxacin was active against 8.6–76.0%, 16.6–88.6%, 87.1–95.6%, 85.1–96.6%, 95.4–100%, 96.8–100%, 17.6–89.9%, 91.2–100%, or 83.3–100%, respectively, of Gram-negative species. Imipenem was active against all Gram-negative aerobes. Methicillin-susceptible *S. aureus* and ampicillin-susceptible enterococci accounted for 37.8% (158 isolates) of staphylococci and 87.2% (102) of enterococci, respectively. Of 396 streptococci, 93.7% (371 isolates) were susceptible to penicillin.

### Prognostic impact of TtAa in the entire cohort

For 3194 patients, the association of clinical variables, in terms of patient demography, bacteremia severity, bacteremia sources, comorbidity severity, and major comorbidities, with 30-day crude mortality was examined in the univariate analysis. The following variables were positively associated with 30-day mortality: the elderly, male, nursing-home residents, inadequate source control, fatal comorbidities (McCabe classification), a critical illness (a Pitt bacteremia score ≥ 4) at ED arrival, bacteremic pneumonia, and comorbidities with malignancies, neurological disorders, or liver cirrhosis (Table 2). In contrast, bacteremia due to urinary tract infections, biliary tract infections, or liver abscess was negatively associated with 30-day mortality. However, nursing-home residents, a critical illness at ED arrival, inadequate source control, bacteremic pneumonia, bacteremia due to urinary or biliary tract infections, fatal comorbidities (McCabe classification), and underlying malignancies or liver cirrhosis were independent prognostic factors in the multivariate regression analysis (Table 2).

**Table 2:** Risk factors of 30-day crude mortality in the entire cohort

Clinical variables	Univariate analysis		Multivariate analysis	
	OR (95% CI)	*P* value	AOR (95% CI)	*P* value
Time-to-appropriate antibiotic (h)*	–	–	1.003 (1.002–1.004)	< 0.001
The elderly	1.25 (1.02–1.54)	0.03	NS	NS
Gender, male	1.54 (1.26–1.88)	< 0.001	NS	NS
Nursing-home residents	2.95 (2.12–4.11)	< 0.001	1.80 (1.18–2.74)	0.006
Inadequate source control during antimicrobial therapy	2.03 (1.285–3.23)	0.002	2.77 (1.55–4.95)	0.001
Pitt bacteremia score ≥ 4 at ED arrival	12.28 (9.86–415.29)	< 0.001	10.40 (8.10–13.36)	< 0.001
Bacteremia sources				
Pneumonia	4.86 (3.89–6.06)	< 0.001	1.76 (1.16–2.39)	< 0.001
Urinary tract	0.21 (0.16–0.29)	< 0.001	0.34 (0.24–0.48)	< 0.001
Biliary tract	0.48 (0.31–0.76)	0.001	0.44 (0.26–0.76)	0.003
Liver abscess	0.42 (0.19–0.91)	0.02	NS	NS
Fatal comorbidities (McCabe classification)	3.43 (2.80–4.20)	< 0.001	2.25 (1.69–3.01)	< 0.001
Comorbidity types				
Malignancies	2.58 (2.12–3.15)	< 0.001	1.86 (1.40–2.47)	< 0.001
Neurological diseases	1.33 (1.07–1.66)	0.01	NS	NS
Liver cirrhosis	1.91 (1.47–2.46)	< 0.001	1.75 (1.26–2.41)	0.001

*AOR* adjusted odds ratio, *CI* confidence interval, *ED* emergency department, *NS* not significant (by backward multivariate regression), *OR* odds ratio

*A continuous variable included in the multivariable logistic regression model

**Fig. 2:**
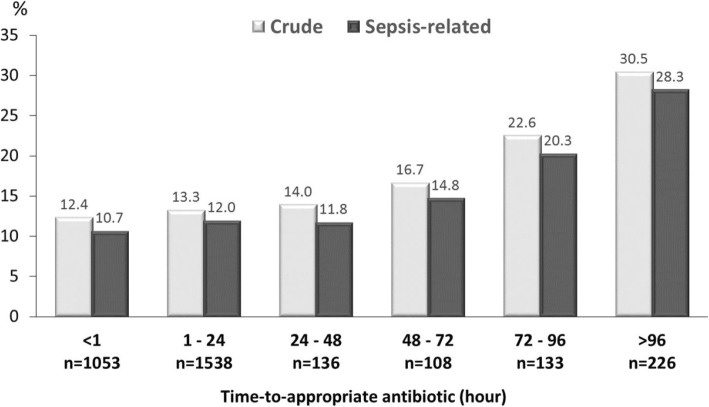
30-day crude and sepsis-related mortality rate in varied periods of time-to-appropriate antibiotic

In the entire cohort, the median (IQR) of TtAa was 2 (1–8) h. A positive TtAa-related trend in 30-day crude (*γ* = 0.919, *P* = 0.01) and sepsis-related (*γ* = 0.909, *P* = 0.01) mortality rate was evidenced in Fig. 2. Notably, the TtAa (measured by hours) remained to be a crucial determinant (adjusted odds ratio [AOR], 1.003; *P* < 0.001) of crude 30-day mortality, after adjustment of nine independent variables (Table 2).

### Prognostic impact of TtAa in critically ill patients

Of 646 patients with a critical illness at ED arrival, the association of clinical variables and 30-day crude mortality was initially examined in a univariate analysis. The following variables were associated with 30-day mortality: fatal comorbidities (McCabe classification), bacteremic pneumonia, primary bacteremia, bacteremia due to urinary or biliary tract infections, and pre-existing malignancies, neurological disorders, or liver cirrhosis (Table 3). In the multivariate regression analysis, there were only three independent variables: fatal comorbidities (McCabe classification), and bacteremia due to urinary or biliary tract infections (Table 4). More importantly, the TtAa (measured by hours) remained to be a crucial predictor (AOR, 1.004; *P* < 0.001) of 30-day mortality, after adjustment of three independent variables.

**Table 3:** Patient demography and clinical characteristics of the critically ill patients (Pitt bacteremia score ≥ 4) surviving at or dying within 30 days after bacteremia onset*

Variables	Patient number (%)			*P* value**
	Total *n* = 646	Death *n* = 302	Survival *n* = 344	
Patient demography				
Age, years, mean ± SD	70.5 ± 15.8	70.6 ± 14.9	70.3 ± 16.5	0.79
Gender, male	370 (57.3)	184 (60.9)	186 (54.1)	0.08
Nursing-home residents	82 (12.7)	39 (12.9)	43 (12.5)	0.88
Time-to-appropriate antibiotic, h, median ± IQR	*2 (1–7)*	*2 (1–15)*	*2 (1–5)*	*< 0.001*
Inadequate source control during antibiotic therapy	26 (4.0)	17 (5.6)	9 (2.6)	0.05
Major bacteremia sources				
Pneumonia	*228 (35.3)*	*133 (44.0)*	*95 (27.6)*	*< 0.001*
Urinary tract	*133 (20.6)*	*26 (8.6)*	*107 (31.1)*	*< 0.001*
Skin and soft-tissue	61 (9.4)	30 (9.9)	31 (9.0)	0.69
Intra-abdominal	59 (9.1)	28 (9.3)	31 (9.0	0.91
Primary bacteremia	*45 (7.0)*	*29 (9.6)*	*16 (4.7)*	*0.01*
Biliary tract	*37 (5.7)*	*11 (3.6)*	*26 (7.6)*	*0.03*
Liver abscess	22 (3.4)	6 (2.0)	16 (4.7)	0.06
Infective endocarditis	18 (2.8)	5 (1.7)	13 (3.8)	0.10
Fatal comorbidities (McCabe classification)	*211 (32.7)*	*124 (41.1)*	*87 (25.3)*	*< 0.001*
Major comorbidities				
Hypertension	308 (47.7)	140 (46.4)	168 (48.8)	0.53
Diabetes mellitus	250 (38.7)	107 (35.4)	143 (41.6)	0.11
Neurological diseases	*235 (36.4)*	*97 (32.1)*	*138 (40.1)*	*0.04*
Malignancies	*220 (34.1)*	*125 (41.4)*	*95 (27.6)*	*< 0.001*
Chronic kidney diseases	114 (17.6)	49 (16.2)	65 (18.9)	0.37
Liver cirrhosis	*76 (11.8)*	*46 (15.2)*	*30 (8.7)*	*0.01*
Coronary artery diseases	64 (9.9)	31 (10.3)	33 (9.6)	0.78

*ED* emergency department, *ICU* intensive care unit, *SD* standard deviation

*Data are number (%) of patients unless otherwise stated

**Indicates the comparison between fatal patients and survivors. Boldface indicates statistical significance, i.e., a *P* value of < 0.05

**Table 4:** Risk factors of 30-day crude mortality in 646 critically ill patients

Clinical variables	Univariate analysis		Multivariate analysis	
	OR (95% CI)	*P* value	AOR (95% CI)	*P* value
Time-to-appropriate antibiotic (h)*	–	–	1.004 (1.003–1.006)	< 0.001
Bacteremia sources				
Primary bacteremia	2.18 (1.16–4.09)	0.01	NS	NS
Pneumonia	2.06 (1.49–2.86)	< 0.001	1.47 (1.00–2.17)	0.05
Urinary tract	0.21 (0.13–0.33)	< 0.001	0.26 (0.16–0.44)	< 0.001
Biliary tract	0.46 (0.22–0.95)	0.03	0.31 (0.14–0.70)	0.005
Fatal comorbidities (McCabe classification)	2.06 (1.47–2.87)	< 0.001	1.79 (1.24–2.57)	0.002
Comorbidity types				
Malignancies	1.85 (1.33–2.57)	< 0.001	NS	NS
Neurological diseases	0.71 (0.51–0.98)	0.04	0.70 (0.49–1.01)	0.06
Liver cirrhosis	1.88 (1.15–3.07)	0.01	NS	NS

*AOR* adjusted odds ratio, *CI* confidence interval, *NS* not significant (by backward multivariate regression), *OR* odds ratio

*A continuous variable included in the multivariable logistic regression model

### Association of time-to-appropriate antibiotics and delayed defervescence in overall febrile patients

Among 2469 febrile patients, the median (IQR) of the TtD, the duration of intravenous antimicrobial administration, and the hospital stay was 4.5 (3.0–9.0), 9.0 (6.5–16.0), and 11.0 (7.0–19.0) days, respectively. Because the mean of the TtD was 6.7 days, a TtD of ≥ 7 days was regarded as delayed defervescence. In univariate analyses, nursing-home residents, a critical illness at ED arrival, polymicrobial bacteremia, bacteremic pneumonia, fatal comorbidities (McCabe classification), and comorbid malignancies were positively associated with delayed defervescence (Table 5). However, bacteremia due to urinary or biliary tract infections were negatively associated with delayed defervescence. In the multivariate regression model, four positive independent predictors (i.e., a critical illness at ED arrival, fatal comorbidities, bacteremic pneumonia, and comorbid malignancies) and two negative independent predictors (i.e., bacteremia due to urinary or biliary tract infections) of delayed defervescence were found (Table 5).

**Table 5:** Risk factors of delayed defervescence (time-to-defervescence ≥ 7 days) in 2469 febrile patients

Clinical variables	Patient number (%)		Univariate analysis		Multivariate analysis	
	Yes, *n* = 904	No, *n* = 1565	OR (95% CI)	*P* value	AOR (95% CI)	*P* value
Time-to-appropriate antibiotic (h)*	–	–	–	–	1.007 (1.005–1.009)	< 0.001
Nursing-home residents	55 (6.1)	57 (3.6)	1.71 (1.17–2.51)	0.005	NS	NS
Pitt bacteremia score ≥ 4 at ED arrival	271 (30.0)	151 (9.6)	4.01 (3.22–5.00)	< 0.001	3.35 (2.64–4.25)	< 0.001
Polymicrobial bacteremia	102 (11.3)	108 (6.9)	1.72 (1.29–2.28)	< 0.001	NS	NS
Bacteremia sources						
Pneumonia	195 (21.6)	101 (6.5)	3.99 (3.09–5.15)	< 0.001	2.05 (1.53–2.73)	< 0.001
Urinary tract	220 (24.3)	673 (43.0)	0.43 (0.36–0.51)	< 0.001	0.49 (0.40–0.60)	< 0.001
Biliary tract	58 (6.4)	165 (10.5)	0.58 (0.43–0.79)	0.001	0.47 (0.33–0.66)	< 0.001
Fatal comorbidities (McCabe classification)	298 (33.0)	308 (19.7)	2.01 (1.67–2.42)	< 0.001	1.39 (1.09–1.76)	0.007
Comorbid malignancies	332 (36.7)	401 (25.6)	1.69 (1.41–2.01)	< 0.001	1.34 (1.07–1.68)	0.01

*AOR* adjusted odds ratio, *CI* confidence interval, *ED* emergency department, *NS* not significant (by backward multivariate regression), *OR* odds ratio

*A continuous variable included in the multivariable logistic regression model

**Fig. 3:**
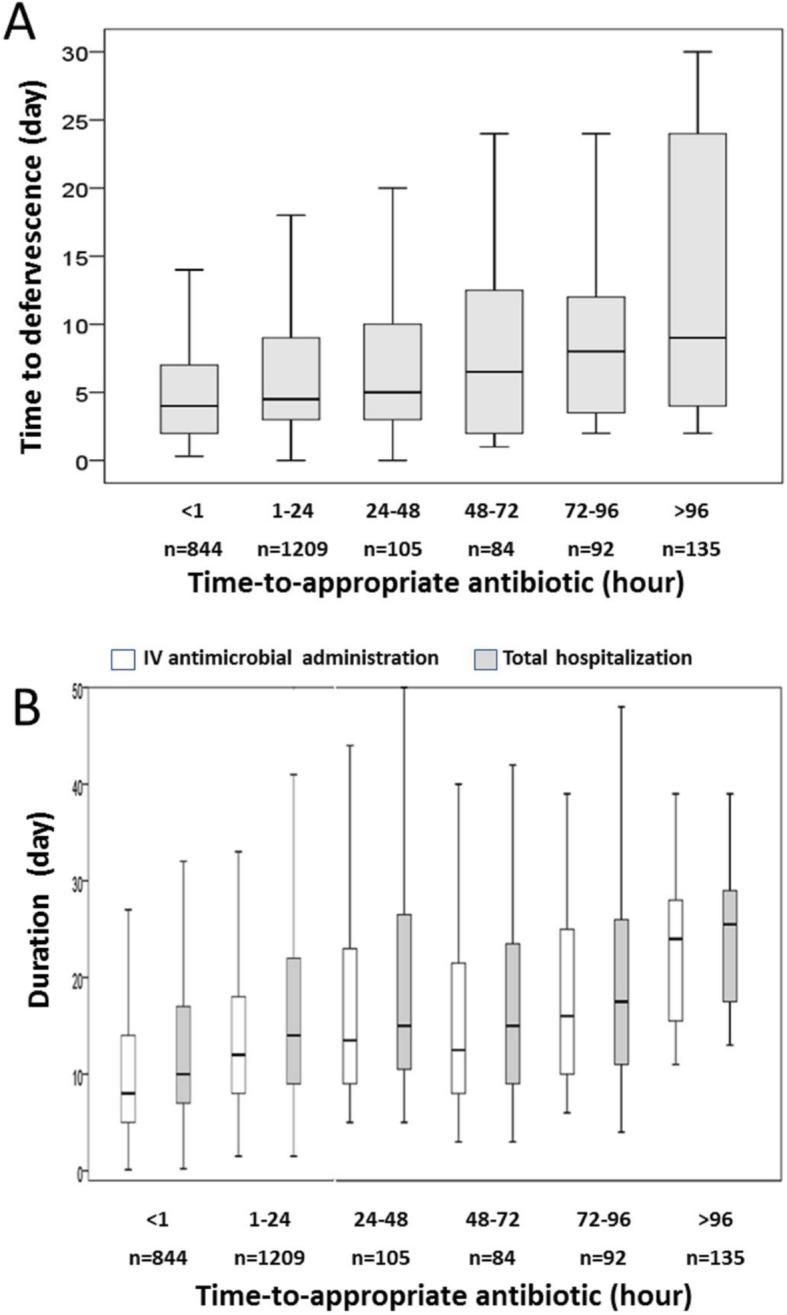
Boxplots of the time-to-defervescence (**a**) and the length of intravenous (IV) antimicrobial administration and total hospitalization (**b**) in febrile patients having the varied categories of time-to-appropriate antibiotic

For febrile patients, there was a linear-by-linear association of the TtAa and the TtD (*γ* = 0.965, *P* = 0.002) (Fig. 3a). Furthermore, a TtAa-related trend in the duration of intravenous antimicrobial administration (*γ* = 0.964, *P* = 0.002) and hospital stay (*γ* = 0.957, *P* = 0.003) was evidenced in Fig. 3b. Of importance, the TtAa (measured by hours) was an independent determinant (AOR, 1.002; *P* = 0.001) of delayed defervescence, after adjustment of six independent predictors (Table 5).

### Impact of time-to-appropriate antibiotics on defervescence in febrile patient subgroups

**Fig. 4:**
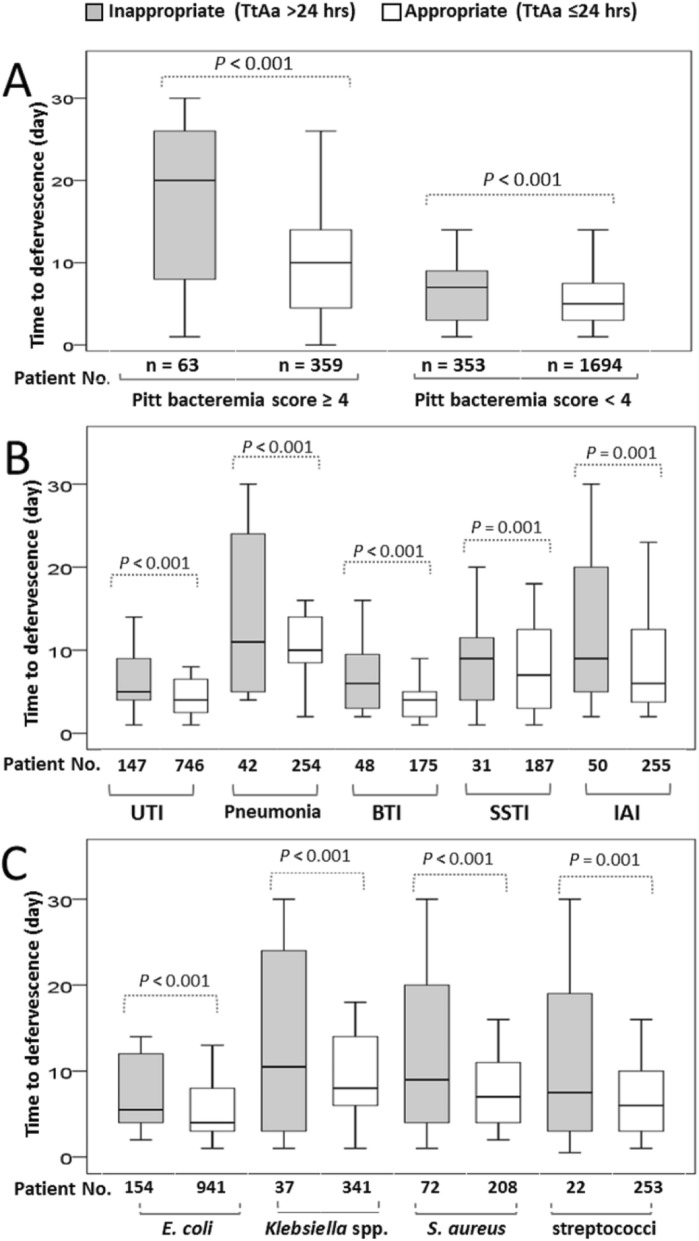
Boxplots for the impact of inappropriate empirical therapy (time-to-appropriate antibiotic [TtAa] > 24 h) on the time-to-defervescence among the patients with the critical or non-critical illness (**a**), different bacteremia sources (**b**), or causative microorganims (**c**)

Of 2469 febrile patients, the mean TtD of 422 critically ill patients at ED arrival was longer than that of 2047 non-critically ill patients (10.2 vs. 6.0 days, *P* < 0.001). Of five leading bacteremic sources, the highest median (IRQ) of the TtD was observed in 296 patients with pneumonia, 10.0 (5.5–14.0) days, followed by 6.0 (3.5–10.0) days in 218 with skin and soft-tissue infections, 5.0 (2.5–9.0) days in 305 with intra-abdominal infections, 4.5 (2.5–7.0) days in 893 with urinary tract infections, and 4.0 (2.0–6.5) days in 223 with biliary tract infections. Of four major causative pathogens, the highest median (IQR) of the TtD was present in 280 patients with *S. aureus* bacteremia, 7.0 (4.5–14.0) days, followed by 5.5 (3.5–10.0) days in 378 with *Klebsiella* bacteremia, 5.0 (2.5–9.0) days in 275 with streptococcal bacteremia, and 4.0 (2.0–7.0) days in 1095 with *E. coli* bacteremia. Regardless of the initial presence of the critical or non-critical illness, bacteremic sources, or causative pathogens, inappropriate empirical antimicrobial therapy (TtAa > 24 h) was significantly related to a longer TtD (Fig. 4).

## Discussion

Bacteremia is associated with substantial morbidity and mortality and thus is typically considered a severe presentation of systemic infections [6]. However, in contrast to the effects of appropriate empirical antibiotic therapy on severe sepsis or septic shock, the potential benefits of appropriate empirical therapy in bacteremia has achieved little consensus. Some studies reported minimal effect [10, 11], whereas some noted a significant reduction of case fatality rates [4, 7–9]. This controversy may be related to the variations in bacteremia severity, comorbidities, immune status, and distribution of causative microorganisms in the study patients [27]. Similar to two studies of bloodstream infections [7, 8], our results support that appropriate initial antimicrobial therapy could reduce short-term mortality in the setting of community-onset bacteremia.

It has been recognized that antimicrobial delays decreased short-term survival in patients with severe sepsis and septic shock [28, 29]. Likewise, the risk of mortality steadily increased; if appropriate, antimicrobial administration was delayed in our patients with community-onset bacteremia. Our cohort demonstrated that each hour of TtAa delay was associated with an increase in 30-day mortality rate of 0.4% in critically ill patients and 0.3% in all enrolled patients. Consequently, for severe bacteremic episodes, the appropriateness of empirical administration should be emphasized. Therefore, in addition to identifying those critically ill cases of bloodstream infections among febrile visitors at the EDs, timely empirical administration of broad-spectrum antimicrobial agents, epidemiological surveillance, and rapid detection of etiological pathogens and antimicrobial susceptibility is crucial for timely administration of appropriate antimicrobials.

The time gap between the initiation of antimicrobial administration and defervescence has been assessed as an outcome parameter in response to antimicrobial therapy in previous investigations of bloodstream infections [12, 30, 31]. Focusing on this outcome vastly linked to the hospital stay, our cohort highlighted the temporal association between the defervescence timing and the appropriateness of empirical antimicrobial therapy was evident in not only non-critically ill cases, but also critically ill ones at initial presentations. Such a therapeutic benefit of timely appropriate therapy also can be noted in the subgroup patients with variable bacteremic sources or causative pathogens. In other words, the association of delayed defervescence and inappropriate empirical antimicrobials was clearly and reasonably demonstrated herein.

Similar to several ED-based investigations of bloodstream infections [32, 33], the bacteremia source of urinary or biliary infections was a protective factor against mortality in our cohort, whereas bacteremia due to pneumonia was a crucial determinant of fatality. From our suspect, major reasons for the differences in the prognoses between these bacteremia sources may have been the different proportions for three most powerful prognostic factors recognized here, in terms of bacteremia severity at initial presentations, comorbidity severity, and adequate source control. For instance, compared with patients with bacteremia due to urosepsis, patients with bacteremic pneumonia had a significantly higher tendency to have a critical illness at ED arrival (228/473, 48.2% vs. 133/1034, 12.9%; *P* < 0.001), severe underlying diseases (i.e., fatal comorbidities) (159/473, 33.6% vs. 173/1034, 16.7%; *P* < 0.001), and inadequate source control (11/473, 2.3% vs. 8/1034, 0.8%; *P* = 0.01).

In addition to the timing of appropriate antimicrobial administration, the role of adequate source control for complicated bloodstream infections was examined herein. Consistent with published studies on severe sepsis [25] and complicated bacteremia [26], we found that inadequate source control was an independent determinant of short-term mortality. Although an association between adequate source control and the TtD was not evident in the literature, it is reasonable to assume individuals with inadequate source control have delayed defervescence. In our cohort, patients with inadequate source control for complicated bacteremia accounted only for the minority (2.4%), but the median TtD was longer in those with inadequate source control than in those who received adequate source control, irrespective of the appropriateness of empirical antimicrobial administration (appropriate, 11 vs. 4 h, *P* < 0.001; inappropriate, 84 vs. 74 h, *P* < 0.001). Therefore, to accurately assess the effect of the TtAa on the TtD, patients with inadequate source control was reasonably excluded from our analyses.

This study possesses several limitations. First, its retrospective and observational nature is the main limitation. However, the study cohort was large, and the record discrepancy was minimized by discussion between the chart reviewers during the capture of clinical information. Valuable information on the interaction between the TtAa and TtD has therefore been provided. Second, antimicrobial therapy is a cornerstone of clinical management for patients with bloodstream infections. Similar to the positive attitude on the appropriateness of empirical antimicrobial administration on patient outcomes [4, 7–11], our study had no detailed information of early goal-directed therapy and the sepsis bundle as covariates. Superior to these established reports, the impact of inadequate source control for complicated bacteremia was firstly highlighted herein. Third, in the analysis of the effect of antimicrobial therapy on patient survival, we excluded those with uncertain outcome. Although the chance that the excluded cases of unknown outcome were too severe to return the healthcare system is substantial, only 148 (4.4%) of 3342 adults with community-onset bacteremia were excluded and trivial selection bias is thus expected. Fourth, to prevent interference from several covariates linked to the TtD, patients with initial afebrile presentation or hypothermia, those with steroid therapy for severe sepsis or adrenal insufficiency, and those without adequate source control were excluded. Therefore, caution should be exercised in interpreting the effects of TtAa delay on the TtD. Finally, because the study hospitals were localized in southern Taiwan, a lack of an independent validation is one of the study limitations, and our results may not be extended to other population with variable infection sources, proportions of antimicrobial-resistant microorganisms, or bacteremia severity. However, several clinical predictors of short-term mortality identified in our cohort, including specific bacteremia sources (such as bacteremic pneumonia) and comorbidities (such as liver cirrhosis and malignancies), are consistent with earlier reports involving ED bacteremia [32, 33]. More importantly, the study findings from our large bacteremia cohort highlight that adults with community-onset bacteremia requires early administration of appropriate antimicrobials to achieve favorable outcomes and therapeutic efficacies, as noted before [16, 28, 29]. Additionally, the beneficial influence of rapid administration of appropriate antimicrobials on defervescence was the novelty for patients with bloodstream infections.

## Conclusions

Regardless of bacteremia severity, early administration of appropriate antimicrobials is a crucial determinant of short-term outcomes in patients with community-onset bacteremia. Additionally, rapid administration of appropriate antimicrobials facilitated defervescence. Therefore, to achieve a favorable outcome and rapid defervescence, epidemiological surveillance, rapid pathogen identification, and the incorporation of broad-spectrum antimicrobial as empirical therapy into an antibiotic stewardship program should be considered, particularly in critically ill patients.

## Data Availability

Available from the corresponding author on reasonable request.

## Abbreviations

AOR: Adjusted odds ratio
CI: Confidence interval
CLSI: Clinical and Laboratory Standards Institute
ED: Emergency department
ICUs: Intensive care units
OR: Odds ratio
SD: Standard deviation
TtAa: Time-to-appropriate antibiotic
TtD: Time-to-defervescence
